# Identification of the main determinants of abdominal aorta size: a screening by Pocket Size Imaging Device

**DOI:** 10.1186/s12947-016-0094-z

**Published:** 2017-01-13

**Authors:** Roberta Esposito, Federica Ilardi, Vincenzo Schiano Lomoriello, Regina Sorrentino, Vincenzo Sellitto, Giuseppe Giugliano, Giovanni Esposito, Bruno Trimarco, Maurizio Galderisi

**Affiliations:** 1 Department of Advanced Biomedical Sciences, Division of Cardiology, Federico II University Hospital, Naples, Italy; 2 Interdepartimental Laboratory of Cardiac Imaging, Federico II University Hospital, Via S. Pansini 5,bld 1, 80131 Naples, Italy

**Keywords:** Abdominal aorta, Pocket size imaging device, Ultrasound, Aging, Cardiovascular risk factors, Coronary artery disease

## Abstract

**Background:**

Ultrasound exam as a screening test for abdominal aorta (AA) can visualize the aorta in 99% of patients and has a sensitivity and specificity approaching 100% in screening settings for aortic aneurysm. Pocket Size Imaging Device (PSID) has a potential value as a screening tool, because of its possible use in several clinical settings. Our aim was to assess the impact of demographics and cardiovascular (CV) risk factors on AA size by using PSID in an outpatient screening.

**Methods:**

Consecutive patients*,* referring for a CV assessment in a 6 months period, were screened. AA was visualized by subcostal view in longitudinal and transverse plans in order to determine the greatest anterior-posterior diameter. After excluding 5 patients with AA aneurysm, 508 outpatients were enrolled. All patients underwent a sequential assessment including clinical history with collection of CV risk factors, physical examination, PSID exam and standard Doppler echoc exam using a 2.5 transducer with harmonic capability, both by expert ultrasound operators, during the same morning. Standard echocardiography operators were blinded on PSID exam and viceversa.

**Results:**

Diagnostic accuracy of AA size by PSID was tested successfully with standard echo machine in a subgroup (*n* = 102) (rho = 0.966, *p* < 0.0001). AA diameter was larger in men than in women and in ≥50 -years old subjects than in those <50 -years old (both *p* < 0.0001). AA was larger in patients with coronary artery disease (CAD) (*p* < 0.0001). By a multivariate model, male sex (*p* < 0.0001), age and body mass index (both *p* < 0.0001), CAD (*p* < 0.01) and heart rate (*p* = 0.018) were independent predictors of AA size (cumulative *R* ^2^ = 0.184, *p* < 0.0001).

**Conclusion:**

PSID is a reliable tool for the screening of determinants of AA size. AA diameter is greater in men and strongly influenced by aging and overweight. CAD may be also associated to increased AA diameter.

## Background

Abdominal aortic aneurysm (AAA) is a localized abnormal dilatation of the aorta defined as a diameter ≥30 mm or a >50% increase of the aortic diameter at the diaphragm [1]. Incidence of AAA is increasing [2] due mainly to life prolongation in the current era. The incidence of AAAs in the general population is about 1.0 to 1.5% [3]. This incidence is particularly high in presence of male gender, advanced age, arterial systemic hypertension, family history of AAA, peripheral artery disease or coronary artery disease (CAD), and/or cerebrovascular disease [4–6]. The most feared complication is rupture, which relates directly to size and is especially frequent in patients with AAA >5.5 cm [7]. AAA rupture entails 85–90% overall mortality, 60% pre-hospital and from 40 to 70% in-hospital (following emergency interventions) [8]. AAAs usually do not produce symptoms and ruptured aneurysms often occur without warning. This comprehensive information highlights the need for an early detection of abdominal aorta (AA) dilatation, together with identification of high-risk patients that could benefit from a screening program.

Ultrasound exam as a screening test for AAA is able to visualize the aorta in 99% of patients and has a sensitivity and specificity approaching 100% in screening settings for AAA [9, 10]. In addition, ultrasound test is non invasive, fast, relatively inexpensive, and without biological risk of radiation. The feasibility of population-based ultrasound screening of AAA has been established through large randomized screening trials [11, 12]. Pocket Size Imaging Device (PSID) is an ultrasound machine not classifiable as a standard echocardiographic machine because of impossibility of calculating chamber volumes and quantifying valvular flow by pulsed or continuous Doppler. It has a potential value as a screening tool [13, 14], because of its possible use in several clinical settings.

The present study was designed to identify the influence of demographic variables and cardiovascular (CV) risk factors on AA size in a screening of outpatient population using PSID and to validate it in comparison to standard transthoracic echo-Doppler exam.

## Methods

Five hundred thirteen consecutive patients*,* referring to Echo-lab of Federico II University hospital for a CV assessment in a 6 months period, were screened. All subjects gave written informed consent and the study was approved by the Institutional Ethical Committee. During the screening, 5 patients with AAA (diameter ≥ 3.0 cm in maximum antero-posterior or latero-lateral dimensions) were identified and excluded from subsequent analysis. The final study population included 508 outpatients (M/F = 305/203). All the patients underwent a sequential assessment including: 1. clinical history with collection of CV risk factors; 2. physical examination; 3. PSID exam (Vscan, GE, Horten, Norway) and 4. standard Doppler echocardiographic exam (Vivid E9 ultrasound scanner, GE, Horten, Norway) using a 2.5 transducer with harmonic capability, both by expert ultrasound operators, during the same morning. Standard echocardiography operators were blinded on PSID exam and viceversa.

Arterial systemic hypertension was diagnosed if systolic blood pressure (BP) exceeded 140 mmHg and/or diastolic BP exceeded 90 mmHg, or if the patient was taking antihypertensive drugs [15]. Hypercholesterolemia was defined as plasma total cholesterol >200 mg/dL, plasma low-density lipoprotein cholesterol >130 mg/dL, or when the patient used lipid-lowering medications [16]. Diabetes mellitus was diagnosed if plasma fasting glucose exceeded 126 mg/dL or if the patient used hypoglycaemic drugs [17]. A history of CAD was documented by hospital records, it including acute coronary syndromes, angina pectoris, previous coronary revascularization procedures and positive inducible ischemia test.

**Fig. 1:**
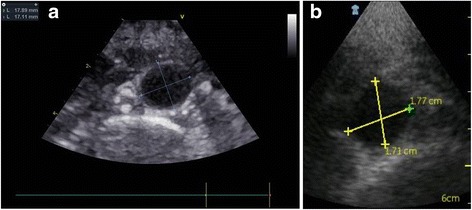
AA visualized in longitudinal and transverse plans from the diaphragm to the bifurcation of the aorta. Antero-posterior and latero-lateral outer diameters were measured in the transverse plane, at the largest portion of infrarenal aorta. The figure shows the good concordance of the two measured diameters between standard echocardiography (panel **a**) and PSID (panel **b**): 17.89 mm versus 1.77 cm and 17.11 mm versus 1.71 cm

AA ultrasound exam was performed using a PSID (unit + probe = 390 g) which provides 2-D, black and white and colour flow images (fixed pulse-repetition frequency and colour-box size), and is connected to a broad-bandwith, phased array probe (1.7–3.8 MHz). The flow sector represents blood flow within an angle of 30°. Videos (automatic autocycle without ECG need) and images can be produced and stored in separate folders, recalled via a gallery function and transferred to hardware by an intermediate docking station. In the present study we utilized an abdominal setting whereas the alternative cardiac/thoracic setting was not applied. AA was visualized using subcostal and abdominal views, with the patient lying supine. No abdominal preparation was required. The entire AA was first visualized in longitudinal and transverse plans from the diaphragm to the aorta bifurcation in order to determine the greatest aortic diameter, which was considered for statistical analyses. Antero-posterior and latero-lateral outer diameters were measured in the transverse plane, at the largest portion of infrarenal aorta [18, 19] (Fig. 1). In a subgroup of 102 patients the diagnostic accuracy of AA size measurements obtained by PSID was tested in comparison with the same measurements taken by a standard echocardiographic machine.

### Statistical analyses

Statistical analyses were performed by SPSS package, release 12 (SPSS Inc, Chicago, Illinois, USA). Data are presented as mean value ± SD. Descriptive statistics were done by one-factor ANOVA (Bonferroni post-hoc test). Intra-class correlation analysis was used to assess agreement of AA size between PSID and standard echo. The null hypothesis was rejected at *p* ≤ 0.05.

## Results

The feasibility of AA measurements by both PSID and standard echo machine was 100%.

**Fig. 2:**
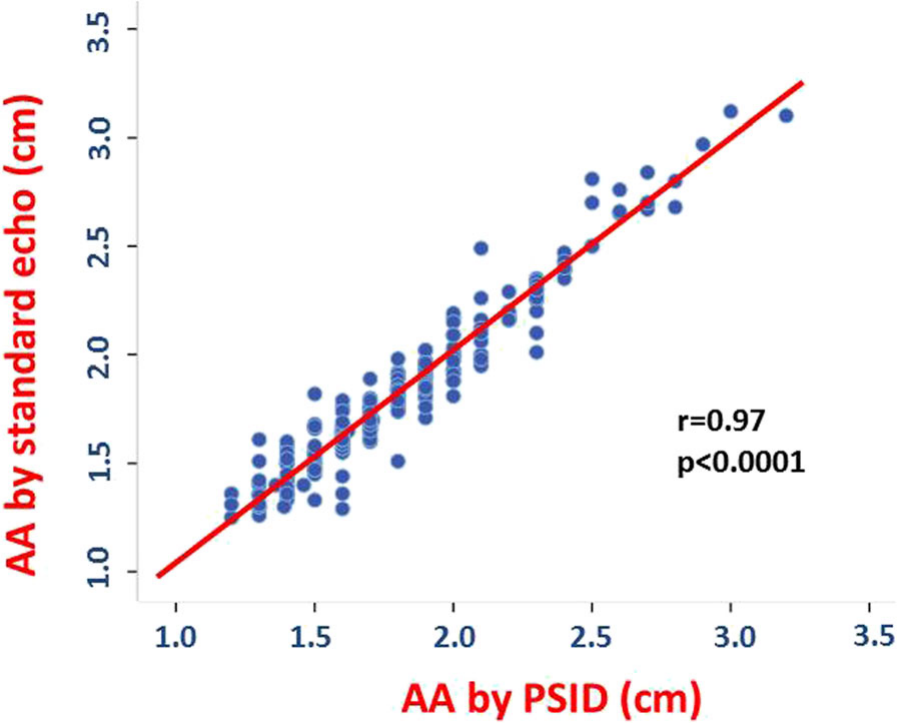
Univariate relation of PSID and standard echocardiographic machine measurements of abdominal aorta (AA) in a subgroup of 102 patients

Figure 2 shows the univariate relation between AA size measured by PSID and that taken by standard echocardiography. The agreement between the two instrumentations was also excellent (rho = 0.966, 95% CI = 0.956–0.974, *p* < 0.0001).

Demographic characteristics and CV risk factors of the study population are listed in Table 1. Of note, hypertensive patients were 64.9% of the study population, the majority being under anti-hypertensive therapy.

**Table 1:** Characteristics of Study Population

Variable	Patients (*n* = 508)
Sex (M/F)	305/203
Age (years)	57.0 ± 15.5
Arterial Hypertension, n (%)	330 (64.9%)
Hypercholesterolemia, n (%)	228 (44.8%)
Type II diabetes mellitus, n (%)	77 (15.1%)
Cigarette smoking, n (%)	115 (22.6%)
Coronary artery disease, n (%)	90 (17.7%)
Anti-hypertensive therapy, n (%)	290 (57.1%)

AA diameter was larger in men (1.84 ± 0.35 cm) than in women (1.65 ± 0.29 cm) (*p* < 0.0001) and in patients with > 50 years (1.80 ± 0.36 cm), compared with patients >50 years old (1.64 ± 0.25 cm) (*p* < 0.0001). Of note, smokers had larger AA diameter in comparison with non smokers (*p* = 0.007) as well as hypercholesterolemic (*p* < 0.01) versus non hypercholesterolemic. Conversely, the presence of both arterial hypertension and diabetes mellitus did not differentiate larger AA diameters. AA was larger also in patients with CAD (1.93 ± 0.43 cm) than in those without (1.72 ± 0.31 cm) (*p* < 0.0001).

**Fig. 3:**
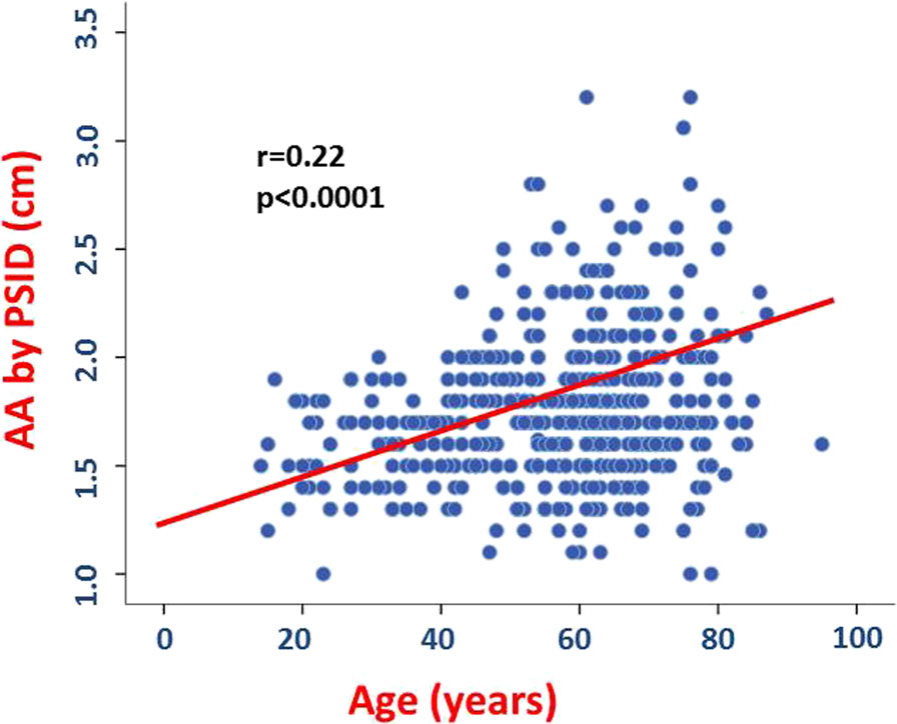
Positive univariate relation between age and abdominal aorta (AA) diameter

**Fig. 4:**
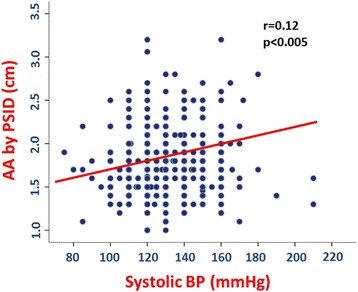
Positive univariate relation between systolic blood pressure (BP) and abdominal aorta (AA) diameter

**Fig. 5:**
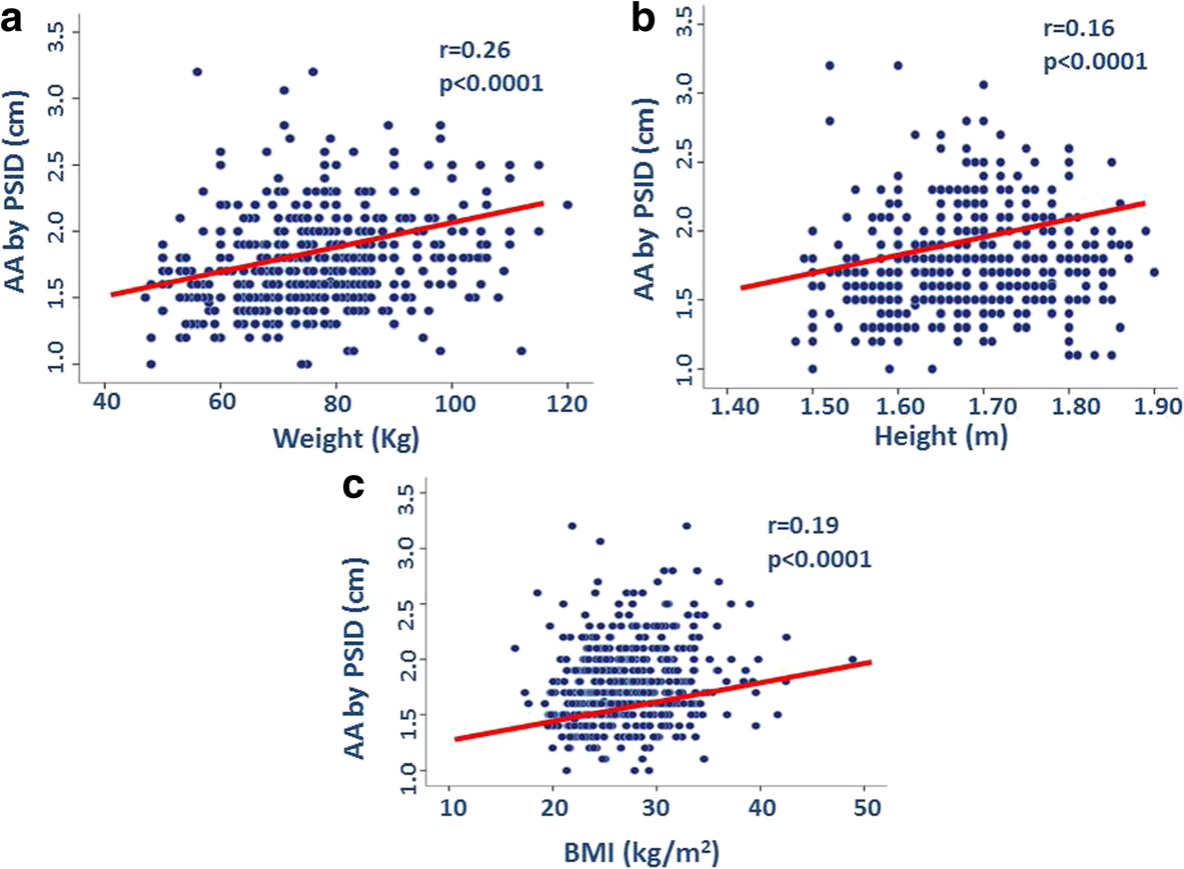
Positive relation of weight (panel **a**), height (panel **b**) and body mass index (BMI) (panel **c**) with abdominal aorta (AA) diameter

In the pooled population, AA diameter was positively related to age (*p* < 0.0001) (Fig. 3), systolic BP (*p* < 0.005) (Fig. 4), mean BP and pulse pressure (both *r* = 0.11, *p* < 0.01), weight, height and body mass index (BMI) (all *p* < 0.0001) (Fig. 5). Diastolic BP and heart rate were not significantly related with AA size.

In a multiple linear regression analysis performed in the pooled population, after adjusting for several confounders, male sex, age and BMI (all *p* < 0.0001), and, with a lesser extent, CAD (*p* < 0.01) and heart rate (*p* = 0.018) were independent predictors of AA size whereas cigarette smoking and hypercholesterolemia did not enter the model (cumulative R^2^ = 0.184, SEE = 0.31 cm, *p* < 0.0001) (Table 2).

**Table 2:** Independent predictors of AA Size by multiple linear regression analysis

Dependent variable	Predictor	Standardized β coefficient	*P* value
AA size	Male sex	0.279	<0.0001
	Age	0.174	<0.0001
	BMI	0.170	<0.0001
	HR	0.099	=0.018
	Systolic BP	0.018	0.671
	Cigarette smoking	0.019	0.654
	Hypercholesterolemia	0.040	0.352
	Coronary artery disease	0.106	<0.01

Cumulative *R* ^2^ = 0.184, SEE = 0.31 cm, *p* < 0.0001

*AA* abdominal aorta, *BP* blood pressure, *BMI* body mass index, *HR* heart rate

## Discussion

The present study demonstrates (1) PSID's excellent feasibility and accuracy in assessing AA size in comparison to standard echocardiography and that (2) by using this tool, male sex, age, body mass index are major independent determinants of AA size, whereas the presence of CAD and increased heart rate should not be underestimated.

PSID is a latest generation, portable device that allows to acquire real-time 2D and colour Doppler images, giving the chance to obtain linear and area measurements of cardiac and vascular structures. Its additional diagnostic value to the simple physical examination has been shown [20–22], particularly in conditions such as evaluation of left ventricular size and function [21–23], right ventricular heart failure [21, 24], mitral valve prolapse [25] and pleural or pericardial effusions [26]. Being a very small unit it offers the potential possibility of an easy and practical use and effectiveness for population screening [13]. The screening for AAA using PSID by experienced physicians has been already proposed as a valuable extension of routine physical examination in vascular patients. It appeared to have a 100% of agreement with a standard ultrasound machine in diagnosing aneurysms in 204 patients hospitalized in a cardiology institute [27]. Another study showed a good diagnostic accuracy in measuring AA size in comparison with standard ultrasound exam in patients referring for acute myocardial infarction in coronary care unit [28]. The present study is in agreement with these findings since we found an excellent concordance between measurements of AA taken by PSID and those obtained by a standard ultrasound machine. Accordingly, PSID can be judged as a valuable tool for detecting AA dilation.

AAA represents still nowadays an important cause of mortality in the western countries [29]. To date, in expert hands, ultrasound exam represents a consolidated tool for AA assessment [30]. Therefore, an effective screening plan could be valuable to prevent extreme AA dilation and rupture and appropriately address high risk patients towards surgery. The importance of an early detection of AA dilatation has been indirectly proven by the observation that AAA and AA rupture can be reasonably excluded in old patients with abdominal pain admitted in emergency department if they had a normal AA size on a previously performed computed tomography or ultrasound exam [31]. A recent study has also shown that a systematic and targeted approach based on CV risk assessment could be very useful to identify undiagnosed cases [32]. The cost-effectiveness of AAA screening programs has been demonstrated in men with >65 years [33]. Even women should be involved in these programs, because, in spite of the lower prevalence, AAA in woman has a higher risk of rupture [34].

By using standard ultrasound machines, determinants of AAA have been more extensively investigated than factors influencing AA size itself. In an unselected population of 742 patients, Bekkers et al. observed that AAA prevalence increased with age, especially in men [35]. In the very large sample size of Tromso study, the prevalence of AAA increased with age, additional factors being represented by smoke, low serum high density lipoprotein cholesterol and antihypertensive therapy [36]. In a meta-analysis of 15 cross-sectional studies, male sex was strongly associated with AAA (OR 5.69), while cigarette smoking (OR 2.41), history of myocardial infarction (OR 2.28) or peripheral vascular disease (OR 2.50) showed moderate associations and arterial hypertension was only weakly associated with AAA (OR 1.33) [37]. The association of obesity with AAA is controversial. Body mass index was not associated with AAA presence and growth in the experiences of Tagaki et al. [38, 39]. However, in a large cohort of 12.203 men who had an ultrasound examination of their AA and filled out a questionnaire including demographic, behavioural and medical variables, AAA was significantly associated with a waist/hip ratio greater than 0.9 [35].

In our study population, we extended the screening to outpatients without overt AAA. By this assessment. male sex, age and BMI were all major independent determinants of AA size, whereas the association of higher heart rate and AA was marginal but significant. Although this latter finding is in disagreement with a cross sectional study showing a negative correlation between heart rate and AA diameter [40], it is conceivable that tachycardia could exert a detrimental effect on AA size [41]. Systolic BP showed a positive univariate relation with AA diameter in our study population but this association disappeared in the multivariate model. Conversely, in a recent study diastolic BP was a risk factor of AAA expansion [42]. The undergoing anti-hypertensive therapy of the majority of our patients (57%, see Table 1) could have blunted the association between increased afterload due to hypertension and AA size of the present study. The independent association of CAD with AA size is consistent with the data of Bekkers et al., who found a significant association of AAA with established coronary and peripheral arterial disease [35] and also with a meta-analysis of 15 cross-sectional studies [37]. Cigarette smoking and hypercholesterolemia were not independently associated with increased AA size, findings which are in disagreement with some previous studies assessing determinants of AAA [36, 37]. It has however to be taken into account that the rate of smoking in our population sample was relatively low and that the present study investigated determinants of AA diameter in earlier stages than that explored in these previous observations on AAA.

### Study limitations

The main limitation of the present study is represented by the fact that we demonstrated the diagnostic capability of PSID in measuring AA and not AAA. However, looking at our correlation between AA data assessed by PSID and standard echo we can suppose that PSID-derived measurements of AAA could be also consistent with those taken by standard echo machine. Another limitation could be considered our lack of correlation data between AA and ascending aorta, an association previously reported by Agricola et al. in patients with known AAA [42]. Finally, PSID derived AA size in the present study was measured by experts in cardiac ultrasound whereas it could be even more important to collect measurements taken by non expert operators.

## Conclusion

The physical examination does not always allow diagnosis of AAA in patients without a very large AA diameter [43]. The findings of the present study demonstrate that the use of a miniaturized and portable device such as PSID could allow to widen the spectrum of patients susceptible of screening, allowing AA visualization also during a routine medical examination. Thus, the physician has the opportunity to complete the evaluation of patients, especially those at higher CV risk, to precociously detect patients with abnormalities of AA size and possibly treat cardiovascular risk factors more aggressively.

## Abbreviations

AA: Abdominal aorta
AAA: Abdominal aorta aneurysm
CAD: Coronary artery disease
CV: Cardiovascular
PSID: Pocket size imaging device
